# The Combined Application of Biofertilizer Alleviates the Continuous Cropping Obstacles of Replanted *Zanthoxylum bungeanum*: A Preliminary Study

**DOI:** 10.3390/plants14060854

**Published:** 2025-03-09

**Authors:** Wei Chen, Shuheng Zhang, Bin Wang, Mengyang Zhang, Dedong Ding, Jing He

**Affiliations:** 1 College of Forestry, Gansu Agricultural University, Lanzhou 730070, China; 18298345663@163.com (W.C.); 18893814674@163.com (S.Z.); wangbin@gsau.edu.cn (B.W.); 18139793655@163.com (M.Z.); ddd3202022@163.com (D.D.); 2 Wolfberry Harmless Cultivation Engineering Research Center of Gansu Province, Lanzhou 730070, China

**Keywords:** *Zanthoxylum bungeanum*, continuous cropping obstacle, biofertilizer, soil phenolic acids, physiological characteristics

## Abstract

In order to effectively alleviate the continuous cropping obstacles in *Zanthoxylum bungeanum* forests, different volume ratios of bio-organic fertilizer and microbial fertilizer were used as remediation agents to investigate their effects on phenolic acids in continuous cropping soil and physiological and biochemical characteristics of replanted *Z. bungeanum* seedlings. The results showed that the combined application of bio-organic fertilizer and microbial fertilizer significantly reduced the contents of gallic acid (GA) and caffeic acid (CA) in continuous cropping soil (*p* < 0.05) and the content of malondialdehyde (MDA) in *Z. bungeanum* leaves and increased the activity of catalase (CAT) in leaves (*p* < 0.05). Compared with the control group without fertilization (T0), the lowest MDA content, the highest superoxide dismutase (SOD), peroxidase (POD) and CAT activities, and the highest accumulation of soluble sugars (SSs) and soluble proteins (SPs) were observed under the T6 treatment (2:1 volume ratio of microbial fertilizer to bio-organic fertilizer). The comprehensive evaluation results of principal components showed that the T6 treatment had the highest comprehensive score. That is, the alleviation effect was most pronounced when the volume ratio of microbial fertilizer and bio-organic fertilizer was 2:1 in combination. This study confirms the potential of biofertilizer combined application technology for repairing continuous cropping obstacles and provides a scalable ecological fertilization scheme for the sustainable cultivation of *Z. bungeanum*.

## 1. Introduction

*Zanthoxylum bungeanum* is a small deciduous tree or shrub of the genus *Zanthoxylum* in the Rutaceae family. *Zanthoxylum bungeanum* has a long history of cultivation in China, rich in germplasm resources and widely distributed, and it is a multi-purpose economic tree species with high economic value for seasoning, spice, oil, and medicinal purposes [1,2,3]. However, with the continuous expansion of planting areas and the extension of planting years, the problem of continuous cropping obstacles in *Z. bungeanum* forests has become increasingly prominent. It is mainly manifested in the decline of soil quality of *Z. bungeanum* forests, the decrease in *Z. bungeanum* yield and quality, and the low survival rate of seedlings after replanting, which seriously restricts the sustainable development of the *Z. bungeanum* industry [4,5,6]. Therefore, how to improve soil quality and alleviate soil continuous cropping obstacles has become a key issue that urgently needs to be addressed in the current cultivation of *Z. bungeanum*.

Soil degradation caused by long-term continuous cropping is an important factor limiting the high quality and high yield of crops, and it is closely related to changes in soil physicochemical properties, microbial community structure, and other factors [7,8]. Continuous cropping obstacles can cause changes in the physicochemical properties of soil, such as nutrient imbalance, a decrease in pH and enzyme activity, and the accumulation of toxic substances, etc. [9,10]. In addition, continuous cropping can also exacerbate the chances of soil-borne diseases, leading to the continuous accumulation of pathogens, a decrease in soil microbial diversity, and a reduction in the number of beneficial microorganisms [11,12]. It has been shown that the continuous accumulation of soil autotoxic substances (mainly phenolic acids) in continuous cropping soil is the key to the occurrence of continuous cropping obstacles [13,14]. Many kinds of allelopathic substances can cause continuous cropping obstacles, and phenolic acids are a class of allelopathic substances with strong activity and have been studied most in recent years. They can enter the soil through pathways such as plant root secretion, aboveground leaching, the decomposition of plant residues and litter, and the release of plant self, thereby affecting plant growth and development [15,16,17]. The excessive accumulation of phenolic acids is manifested as the inhibition of seed germination and ion uptake in seedlings, the disruption of plant cell membrane structure, the impact on enzyme metabolism, hormone metabolism, and interference with physiological and biochemical processes such as DNA replication and RNA transcription [18]. Therefore, it is of great theoretical and practical significance to study the change in phenolic acid in the soil to alleviate or overcome the obstacles of continuous cropping.

Currently, the measures that can be used to alleviate the obstacles of continuous cropping include reasonable crop rotation and intercropping, soil improvement, the scientific and reasonable ratio of fertilization, and the selection of disease-resistant varieties [19,20,21,22]. Especially, in recent years, the application of soil amendments to alleviate allelopathic autotoxicity and overcome continuous cropping obstacles has become a research focus. Microbial fertilizers are widely used in production because they contain a large number of beneficial microbial flora [23,24]. Among them, bio-organic fertilizers are mainly composed of functional microorganisms and organic materials derived from animal and plant residues (such as livestock and poultry manure, crop straw, etc.) that have been treated harmlessly and decomposed, combining the excellent effects of microbial fertilizers and organic fertilizers. Moreover, bio-organic fertilizers have the characteristics of comprehensive nutrient balance and long-lasting fertilizer efficiency, and they are rich in functional microorganisms, which have been used in soil nutrient activation, plant growth promotion, and soil-borne disease prevention and control [24,25,26]. Microbial agents are live microbial preparations made through special processes using beneficial microbial flora, which have the effect of increasing soil fertility and improving the soil microbial structure [27,28]. Both of them can improve soil and rhizosphere microbial ecology, enhance plant resistance, and have good development and application prospects in alleviating plant continuous cropping obstacles [23,29].

Currently, research on *Z. bungeanum* mainly focuses on phenolic chemotype characteristics, anatomy and histochemistry [30], the biodiversity and antifungal activity of endophytic fungi [31], the isolation and antioxidant activity of polysaccharides [1,3], phytochemistry, and health functions [2], etc. However, there are few reports on the effects of biofertilizers applied in combination on soil phenolic acids and the physiological characteristics of *Z. bungeanum* in continuous cropping obstacles. Our team previously treated continuous cropping soil with bio-organic fertilizers and microbial fertilizers and explored the effects of biofertilizer mixing on the growth and photosynthesis of continuous cropping *Z. bungeanum* [6]. However, the effects on the physiological and biochemical characteristics of replanted *Z. bungeanum* and soil allelopathic substances are still unclear. The relationship between soil phenolic acids and the physiological metabolism of *Z. bungeanum* seedlings is also unknown. Therefore, in this study, different volume ratios of bio-organic fertilizers and microbial fertilizers were used as remediation agents to investigate their effects on soil phenolic acids and the physiology and biochemistry of replanted *Z. bungeanum* seedlings, with a view to provide a theoretical basis for alleviating the continuous cropping obstacles in *Z. bungeanum* forests.

## 2. Results

### 2.1. The Effect of Combined Application of Biofertilizers on the Content of Phenolic Acids in Replanted Z. bungeanum Soil

**Figure 1:**
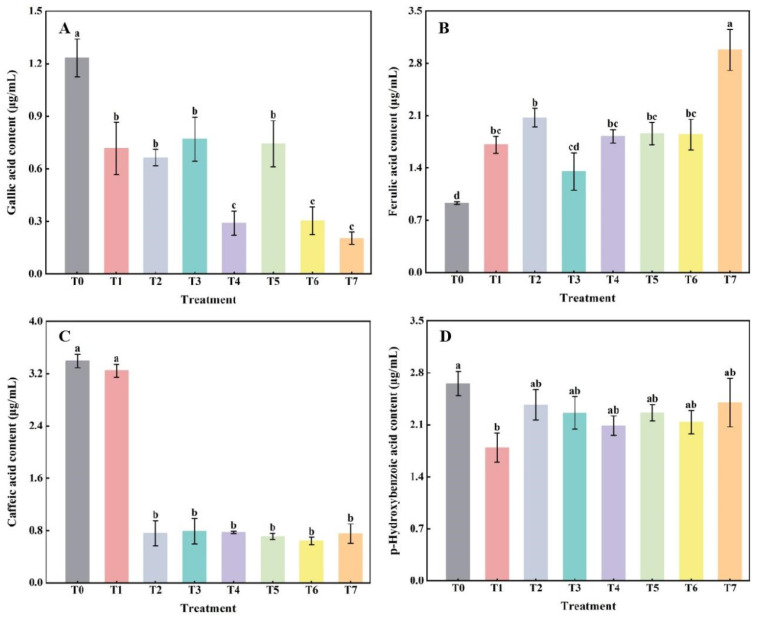
The effect of the combined application of biofertilizers on the content of (**A**) gallic acid, (**B**) ferulic acid, (**C**) caffeic acid, and (**D**) p-hydroxybenzoic acid in replanted *Z. bungeanum* soil. T0, no fertilization control; T1, 10 g·L^−1^ microbial fertilizer; T2, 100 g·L^−1^ bio-organic fertilizer; T3–T7 denote that the volume ratio of microbial fertilizer to bio-organic fertilizer is 1:2, 1:4, 1:1, 2:1, and 4:1, respectively. Different lowercase letters on the bar chart indicate significant differences between different treatments (*p* < 0.05).

Compared with the control (T0), the content of gallic acid was significantly decreased (*p* < 0.05) after fertilization treatment (T1–T7) (Figure 1A). The most pronounced decreases were at T4, T6, and T7. On the contrary, the content of ferulic acid in different fertilization treatments increased compared with T0. Except for T3, which showed no significant difference from T0, all other treatments showed significant differences from T0 (*p* < 0.05). The content of ferulic acid treated with T7 was the highest (Figure 1B). Except for no significant difference in caffeic acid content between the T1 and T0 treatments, the other fertilization treatments (T2 to T7) were significantly lower (*p* < 0.05) than that of the T0 treatment. There were no significant differences between treatments T2 to T7, but all were significantly lower than the T1 treatment (Figure 1C). The p-hydroxybenzoic acid content in the T1 treatment was significantly lower than that of the T0 treatment, while the other fertilization treatments showed a decrease in comparison to T0, but the difference was not significant (Figure 1D).

Taken together, the average proportion of the four phenolic acids in all treatments showed p-hydroxybenzoic acid > ferulic acid > caffeic acid > gallic acid. The average percentage of p-hydroxybenzoic acid in the four phenolic acids was 37.02%, and gallic acid was 10.14%. It can be seen that gallic acid was low overall in the soil, and p-hydroxybenzoic acid was relatively high (Figure 1).

### 2.2. The Effect of Combined Application of Biofertilizers on Membrane Peroxidation Products and Defense Enzyme Activities in Replanted Z. bungeanum

**Figure 2:**
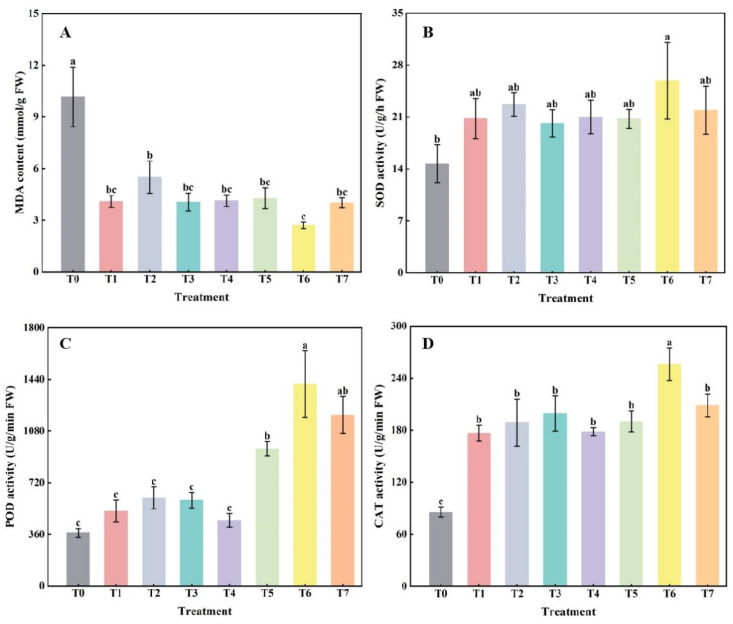
The effect of the combined application of biofertilizers on the MDA content (**A**), the activities of SOD (**B**), POD (**C**), and CAT (**D**) in replanted *Z. bungeanum*. T0, no fertilization control; T1, 10 g·L^−1^ microbial fertilizer; T2, 100 g·L^−1^ bio-organic fertilizer; T3–T7 denote that the volume ratio of microbial fertilizer to bio-organic fertilizer is 1:2, 1:4, 1:1, 2:1, and 4:1, respectively. Different lowercase letters on the bar chart indicate significant differences between different treatments (*p* < 0.05).

The MDA content of *Z. bungeanum* leaves was significantly decreased (*p* < 0.05) in all fertilizer treatments compared with the no-fertilizer control (T0). There was no significant change in MDA content among different fertilization treatments, and only the T6 treatment was significantly lower than the T2 treatment (Figure 2A). After fertilization treatments, only the SOD activity of *Z. bungeanum* leaves in T6 treatment was significantly higher than that of control (T0) and other fertilization treatments, while there was no significant difference among the other treatments (Figure 2B). When the ratio of microbial fertilizer to bio-organic fertilizer was ≥1 (T5 to T7), POD activity was significantly increased (*p* < 0.05). There were no significant changes between treatments from T0 to T4 (Figure 2C). The CAT activities of *Z. bungeanum* leaves were significantly increased (*p* < 0.05) by all fertilization treatments, and there were no significant differences among the other fertilization treatments (Figure 2D).

### 2.3. The Effect of Combined Application of Biofertilizers on the Accumulation of Osmoregulatory Substances in Replanted Z. bungeanum

**Figure 3:**
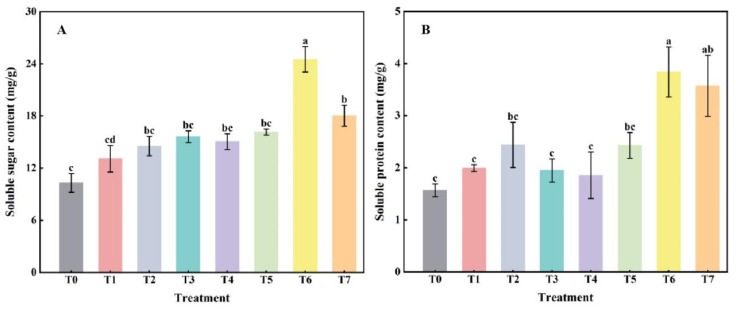
The effect of combined application of biofertilizers on the content of soluble sugar (**A**) and soluble protein (**B**) in replanted *Z. bungeanum*. T0, no fertilization control; T1, 10 g·L^−1^ microbial fertilizer; T2, 100 g·L^−1^ bio-organic fertilizer; T3–T7 denote that the volume ratio of microbial fertilizer to bio-organic fertilizer is 1:2, 1:4, 1:1, 2:1, and 4:1, respectively. Different lowercase letters on the bar chart indicate significant differences between different treatments (*p* < 0.05).

Compared with the no-fertilization control (T0), the contents of SS (Figure 3A) and SP (Figure 3B) in *Z. bungeanum* leaves increased to some extent under different fertilization treatments, but there was no significant difference between T1–T5 treatments and T0, whereas the difference between the T6–T7 treatments and T0 was significant (*p* < 0.05; Figure 3). In addition, there was no significant difference among the treatments from T1 to T5.

### 2.4. The Effect of Combined Application of Biofertilizers on NR Activity in Replanted Z. bungeanum

**Figure 4:**
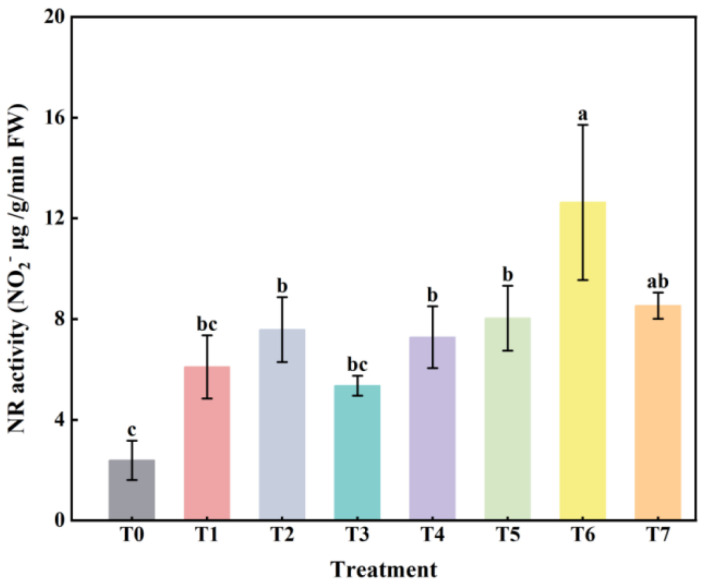
The effect of combined application of biofertilizers on NR activity in replanted *Z. bungeanum*. T0, no fertilization control; T1, 10 g·L^−1^ microbial fertilizer; T2, 100 g·L^−1^ bio-organic fertilizer; T3–T7 denote that the volume ratio of microbial fertilizer to bio-organic fertilizer is 1:2, 1:4, 1:1, 2:1, and 4:1, respectively. Different lowercase letters on the bar chart indicate significant differences between different treatments (*p* < 0.05).

Compared with the no-fertilization control (T0), the NR activity of *Z. bungeanum* leaves increased under different fertilization treatments (Figure 4). Among them, there was no significant difference between T1, T3 treatments, and T0, while the other treatments had significantly higher NR activity than the T0 treatment (*p* < 0.05). Except for T6, there was no significant difference among the fertilization treatments.

### 2.5. Correlation Analysis Between Physiological and Biochemical Indicators of Replanted Z. bungeanum and Soil Phenolic Acids

**Figure 5:**
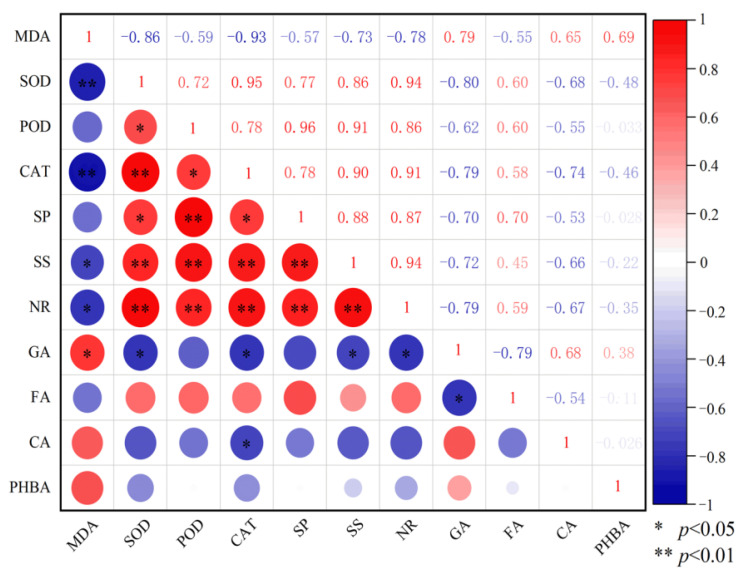
Correlation analysis between physiological and biochemical indicators of replanted *Z. bungeanum* and soil phenolic acids. MDA, malondialdehyde; SOD, superoxide dismutase; POD, peroxidase; CAT, catalase; SP, soluble protein; SS, soluble sugar; NR, nitrate reductase; GA, gallic acid; FA, ferulic acid; CA, caffeic acid; and PHBA, p-hydroxybenzoic acid.

The correlation analysis results between various indicators are shown in Figure 5. MDA showed a highly significant negative correlation with SOD and CAT (*p* < 0.01), a significant negative correlation with SS and NR (*p* < 0.05), and a significant positive correlation with gallic acid (*p* < 0.05). SOD showed a highly significant positive correlation with CAT, SS, and NR (*p* < 0.01), a significant positive correlation with POD and SP (*p* < 0.05), and a significant negative correlation with gallic acid (*p* < 0.05). POD was extremely significantly positively correlated with SP, SS, and NR (*p* < 0.01) and significantly positively correlated with CAT (*p* < 0.05). CAT showed a highly significant positive correlation with SS and NR (*p* < 0.01), a significant positive correlation with SP (*p* < 0.05), and a significant negative correlation with gallic acid and caffeic acid (*p* < 0.05). SP was significantly positively correlated with SS and NR (*p* < 0.01). SS showed a highly significant positive correlation with NR (*p* < 0.01) and a significant negative correlation with gallic acid (*p* < 0.05). NR was significantly negatively correlated with gallic acid (*p* < 0.05). There was a significant negative correlation between gallic acid and ferulic acid (*p* < 0.05).

### 2.6. Principal Component Analysis

Principal component analysis was conducted on the measured indicators, and two principal components with eigenvalues greater than 1 were extracted, with eigenvalues of 7.663 and 1.439 (Table 1), respectively. The cumulative variance contribution rate of the two principal components reached 82.74% (Table 1), indicating that the extracted two principal components covered most of the information of the indicators.

Based on the scores of the two principal components, the corresponding coefficients were calculated to obtain the relevant linear equations:

Y1 = 0.348X1 + 0.347X2 + 0.344X3 + 0.332X4 + 0.315X5 − 0.315X6 + 0.307X7 − 0.294X8 − 0.264X9 + 0.255X10 − 0.123X11

Y2 = −0.117X1 + 0.029X2 − 0.121X3 + 0.117X4 + 0.336X5 + 0.368X6 + 0.323X7 + 0.058X8 − 0.127X9 + 0.084X10 + 0.760X11

Combining the variance contribution rates of the two principal components, the linear equation for the total score was obtained as Y = 0.6966×Y1 + 0.1308×Y2.

The calculation results are shown in Table 2. It can be concluded that the comprehensive restoration effect between different fertilization ratios was T6 > T7 > T5 > T2 > T4 > T3 > T1 > T0.

## 3. Discussion

### 3.1. The Effect of Combined Application of Biofertilizers on Phenolic Acids in Replanted Z. bungeanum Soil

There are many reasons for continuous cropping obstacles, such as the deterioration of soil physical and chemical properties, an increase in harmful microorganisms, the imbalance of microbial flora structure, and the accumulation of autotoxic substances [32]. Among them, the accumulation of phenolic acids in soil is an important cause of continuous cropping obstacles [33]. In this study, four phenolic acids, gallic acid, ferulic acid, caffeic acid, and p-hydroxybenzoic acid, which are widely present in continuous cropping soils, were determined to verify whether the application of two types of biofertilizers affected the content of major allelopathic substances in soil. The results showed that biofertilizers applied in combination could significantly reduce the contents of gallic acid (Figure 1A) and caffeic acid (Figure 1C) in *Z. bungeanum* continuous cropping soil. Shi et al. [34] found that inoculation with *Funneliformis mosseae* inhibited the production of phenolic acid autotoxic substances in continuous cropping soybean during the filling period, similar to the results of this study. The above results indicated that the combined application of the two fertilizers had a certain inhibitory effect on the secretion and release of allelopathic substances in the rhizosphere of *Z. bungeanum*. On the other hand, it may also be due to the improvement of the physical and chemical properties of continuous cropping soil and the microbial environment in the root zone of seedlings by biofertilizers, which leads to an increase in the types and quantities of beneficial microorganisms, thereby accelerating the decomposition and transformation of phenolic acids [33]. In any case, we only observed a reduction in the concentration of phenolic acids under the application of biofertilizers in our study, but the mechanism that caused the reduction is only speculative. Whether this reduction is caused by microbial activity in biofertilizers, changes in soil chemistry driven by microbial activity, or plant-microbe interactions, will be the focus of future mechanistic studies.

In this study, compared with no fertilization control (T0), the content of ferulic acid in soil was significantly increased after the application of biofertilizer, while the content of p-hydroxybenzoic acid was slightly decreased, but the difference was not significant. We speculated that this may be the result of the combined effects of the metabolic activity of microorganisms in fertilizers, interactions among phenolic acids, and plant allelopathy. Chen et al. [33] found that the application of the *Bacillus velezensis* XC1 microbial fertilizer resulted in a significant decrease in ferulic acid content in apple continuous cropping soil, which was inconsistent with the results of this study. This may be related to the differences in the functionality of the fertilizer. For example, two types of fertilizers were applied in this study: bio-organic and microbial fertilizers, with the former being rich in organic matter and a variety of nutrients, and the latter focusing on the introduction of specific beneficial microorganisms, whereas Chen et al. [33] mainly applied *B. velezensis* XC1 bacterial fertilizer. On the other hand, it may also be due to the differences in the research objects and the intensity of plant allelopathy.

In summary, the average proportion of p-hydroxybenzoic acid among the four phenolic acids was 37.02%, and that of gallic acid was 10.14%. That is, the overall content of gallic acid in soil was low, while the content of p-hydroxybenzoic acid was relatively high, a result similar to that analyzed by Zhang et al. [35]. Soil phenolic acids were dominated by p-hydroxybenzoic acid and caffeic acid in the T0 treatment (25-year continuous cropping soil) but were dominated by p-hydroxybenzoic acid and ferulic acid after biofertilizer treatments. This indicated that ferulic acid and caffeic acid were the most sensitive to changes among the four phenolic acids after the application of microbial and bio-organic fertilizers, and similar results were obtained by Pedersen et al. [36].

Correlation analysis showed that gallic acid was significantly negatively correlated with ferulic acid (*p* < 0.05) but positively correlated with caffeic acid and p-hydroxybenzoic acid (*p* > 0.05), indicating that there were differences in phenolic acid accumulation characteristics in the continuous cropping soil of *Z. bungeanum* and that there were different degrees of promotion or inhibition effects among phenolic acids, as the mode of action may depend on the phenolic acid species. Xie et al. [37] also concluded that there was an interaction effect among phenolic acids, which was consistent with the results of this study.

### 3.2. The Effect of Combined Application of Biofertilizers on Physiological and Biochemical Characteristics of Replanted Z. bungeanum

As important allelopathic substances in continuous cropping systems, phenolic acids can affect plant production by causing changes in the structure of rhizosphere microbial communities through direct or indirect autotoxicity, as well as affect plant growth by interfering with the plant’s membrane system and antioxidant enzyme system, etc. [33,37,38]. In this study, the content of gallic acid was significantly negatively correlated with the activities of SOD and CAT (*p* < 0.05), and the content of caffeic acid was significantly negatively correlated with the activity of CAT (*p* < 0.05), which also confirmed this. It has been found that, when phenolic acids accumulate to a certain extent in soil, reactive oxygen species in plants increase sharply, causing a series of physiological and biochemical reactions, resulting in a significant increase in MDA content, which in turn results in the disruption of the plasma membrane system and osmotic homeostasis [39,40]. This was consistent with the results in this study that gallic acid was significantly positively correlated with MDA (*p* < 0.05) and negatively correlated with SS (*p* < 0.05).

To scavenge reactive oxygen species in plants, they form an antioxidant defense enzyme system consisting of SOD, CAT, and POD, among others, which play a crucial role in scavenging free radicals and protecting cells from oxidative damage [41]. In addition, an increase in osmoregulatory substances is essential for plants to maintain cell turgor pressure and stabilize physiological functions under adversity [42,43]. In this study, compared with no-fertilization control (T0), the MDA content of *Z. bungeanum* leaves was significantly decreased (*p* < 0.05), and CAT activity was significantly increased (*p* < 0.05) after fertilization treatments. Moreover, under T6 treatment, MDA content was the lowest, SOD, POD, and CAT activities were the highest (Figure 2), and SS and SP accumulated the most (Figure 3). In other words, when the volume ratio of microbial fertilizer to bio-organic fertilizer was 2:1, the antioxidant enzyme activity of the leaves of *Z. bungeanum* seedlings could be significantly improved, the accumulation of osmoregulatory substances could be increased, and the degree of membrane peroxidation could be reduced. This may be because, on the one hand, biofertilizers applied in combination can reduce the content of phenolic acids such as gallic acid and caffeic acid in continuous cropping soils, thereby reducing the damage caused by phenolic acids to seedling roots. On the other hand, it may also be that biofertilizers indirectly enhance plant metabolism by promoting root uptake and improving the soil microenvironment, allowing plants to mobilize antioxidant defenses more efficiently and improve self-repair and adaptation when faced with continuous cropping obstacles. Anli et al. [44] found that, compared with the control, dual inoculation (plant growth-promoting rhizobacteria and arbuscular mycorrhizal fungi) and composting treatments significantly increased the sugar and protein contents as well as antioxidant enzyme activities in the leaves of *Phoenix dactylifera*, especially under drought stress. Li et al. [45] reported that SOD and CAT activities, as well as the levels of SS and proline in *Brassica rapa*, were also increased after the application of biofertilizers, respectively. These results are similar to those in this study.

NR is a key indicator reflecting the nitrogen metabolism ability of plants [46]. In this study, the NR activity of *Z. bungeanum* leaves increased under different fertilization treatments compared with the no-fertilization control (T0), with the highest NR activity in the T6 treatment (Figure 4). This result was similar to that of Cordeiro et al. [47], indicating that the application of biofertilizers could effectively promote nitrogen metabolism and nutrient uptake in *Z. bungeanum* seedlings. When the volume ratio of microbial fertilizer to bio-organic fertilizer was 2:1, the nitrogen cycle ability of *Z. bungeanum* seedlings was enhanced, which effectively alleviated the continuous cropping obstacles. In addition, correlation analysis revealed that SOD, POD, CAT, and NR were significantly or highly significantly positively correlated with each other (*p* < 0.05 or 0.01). A positive correlation between antioxidant enzyme activities and nitrogen-metabolizing enzyme activities was also found in a study conducted by Li et al. [48]. This suggested that plants can jointly resist adverse external conditions through the synergistic effects among defense enzymes or nitrogen metabolism enzymes. Specifically, SOD, POD, and CAT, as key enzymes in the plant antioxidant system, mitigate cellular damage from oxidative stress by effectively scavenging reactive oxygen species and may provide a relatively stable intracellular environment for nitrogen-metabolizing enzymes such as NR. The enhancement of NR activity, on the other hand, directly promotes the conversion of nitrate to nitrite, which in turn accelerates the nitrogen assimilation process and provides the necessary nitrogen source for plant growth and development.

### 3.3. Research Prospects

The results of this study deepened our understanding of phenolic acid interactions in continuous cropping soils to some extent and provided potential application pathways and actionable strategies for agricultural production and practice management. By translating these research results into specific agricultural management measures, we are expected to effectively alleviate the continuous cropping obstacles of crops such as *Z. bungeanum* in actual production and promote sustainable agricultural development. In the future, strengthening cooperation with agricultural technology extension departments and rapidly transforming research results into technical guidelines usable by farmers will be an important direction for promoting progress in this field.

However, taken together, our study also has some limitations. In this study, only four common phenolic acids were determined, while other phenolic acids were not quantified. Therefore, the observations were only within a specific range, with some selective effects, and further studies on other phenolic acids are necessary. In addition, this study only considered the effects of different fertilizers applied in combination in the second year and not the effects of basal fertilizer applied in the first year, and there is a need to further validate the long-term effects of basal fertilizer on soil microbiology.

## 4. Materials and Methods

### 4.1. Overview of the Study Area

This experiment was conducted at the Economic Forestry Teaching and Research Practice Base of the College of Forestry, Gansu Agricultural University. The study area is located at 36°03′ N and 103°40′ E, with a typical temperate continental climate, with an average annual temperature of 10.3 °C, an average annual precipitation of 327 mm, and an average annual sunshine time of 2446 h.

### 4.2. Test Materials

The test plants were well grown, healthy annual “Dahongpao” *Z. bungeanum* seedlings, purchased from Qin’an County, Tianshui City, Gansu Province. The test soil was taken from a *Z. bungeanum* plantation in Qin’an County, Tianshui City with 25 years of continuous cropping, with a low level of soil fertility, and the basic fertility indicators are shown in Table 3.

The tested biofertilizers included bio-organic fertilizer (solid) and microbial fertilizer (solid). Bio-organic fertilizer was produced by Shandong Ruipu Biotechnology Co., Ltd. (Weifang, China), the active ingredients are organic matter ≥ 30%, N + P_2_O_5_ + K_2_O ≥ 6% (mass fraction), alginic acid ≥ 15%, crude protein ≥ 9% (mass fraction), chelated trace elements, etc., and the effective viable bacteria ≥200 million/g. Microbial fertilizer was produced by Gansu Daxing Agricultural Science and Technology Development Co., Ltd. (Lanzhou, China), and the main ingredients are *Bacillus subtilis*, *Bacillus licheniformis*, rhizobium, humic acid, trace elements, potassium indolebutyrate, sodium naphthalene acetate, with an effective viable bacteria ≥ 200 million/g, N + P_2_O_5_ + K_2_O ≥ 8% (mass fraction).

### 4.3. Experimental Design

In early June 2019, the tested continuous cropping soil was placed in plastic flower pots (32 cm in inner diameter, 25 cm in height), with 15 kg of soil per pot. Annual *Z. bungeanum* seedlings with uniform growth were selected and transplanted into pots, 1 plant per pot. The control was not fertilized, and the other treatments were fertilized with 50 g·L^−1^ of bio-organic fertilizer. The seedlings were normally supplied with water, and measures such as sun protection, frost prevention, and rain prevention should be taken in the early stage of cultivation. To improve the survival rate of seedlings, the application of bio-organic fertilizer in the form of topdressing was continued in the soil after 30 d of equilibration. Before application, the bio-organic fertilizer solids were ground into powder, weighed according to the demand, and dissolved in water. Fertilizer was applied by spraying, every 30 d, with topdressing concentrations of 50 g·L^−1^, 80 g·L^−1^, and 100 g·L^−1^, respectively, at a rate of 500 mL per plant. The application period ended in early October (defoliation period), and the seedlings were finally moved indoors to ensure a safe winter.

After the seedlings were overwintered, a topdressing was carried out in early June 2020, and the spraying was carried out in the morning when there was no wind or rain. The experiment consisted of 8 treatments, including no fertilization control (T0), 10 g·L^−1^ microbial fertilizer (T1), 100 g·L^−1^ bio-organic fertilizer (T2), and volume ratios of microbial fertilizer to bio-organic fertilizer were 1:2 (T3), 1:4 (T4), 1:1 (T5), 2:1 (T6), and 4:1 (T7), respectively. There were 6 replications per treatment. The gradient settings followed the following principles: the T1 and T2 single application treatments were used to determine the biological effects of a single fertilizer, while T3–T7 treatments covered a gradient range from 1:4 to 4:1 with a view to obtain the optimum fertilizer application ratio. The concentration of microbial fertilizer (10 g·L^−1^), as well as the concentration of bio-organic fertilizer (100 g·L^−1^), was obtained by screening based on the results of the preliminary experiments (fertilization treatment with different concentrations of the same fertilizer type). In the case of mixed treatments (T3–T7), the mother liquor was first configured with the concentrations obtained by the screening of the two fertilizers, and, then, the respective mother liquors were mixed according to pre-set different volume ratios before preparing. The total volume of fertilization for each plant was 500 mL, with a total of two fertilizations. Normal management was carried out after fertilizer application, watering every two days (500 mL), and regular weeding was carried out to ensure the normal growth of seedlings. After 30 d of fertilization, samples were collected for determination.

### 4.4. Test Methods

#### 4.4.1. Determination of Phenolic Acids in Soil

Soil samples were collected using the “shake method”. The top layer of soil was first removed with a tool, and, then, the whole plant was carefully dug out and gently shaken off. After the rhizosphere soil was completely shaken off, it was naturally air-dried, and the impurities such as roots and stones were removed from the soil and sieved through a 20-mesh sieve, then collected and brought back to the laboratory and stored at 4 °C for reserve.

The extraction of phenolic acids from soil followed the method proposed by Tian et al. [36]. We took 25 g of fresh soil, added 25 mL of a 1 mol·L^−1^ NaOH solution, stood for 24 h, shook on a 210 r·min^−1^ shaker for 30 min, and then centrifuged at 8000× *g* for 10 min. We separated the supernatant and adjusted the pH of the solution to 2.5 with 12 mol·L^−1^ of HCl to precipitate humic acid. After standing for 2 h, we centrifuged at 8000× *g* for 10 min and stored the separated supernatant at 4 °C for determination.

The determination of soil phenolic acids was carried out using a quaternary gradient ultra-high-performance liquid chromatography (ACQUITY Arc, Waters Corporation, Milford, MA, USA) with reference to the methods of Tian et al. [49] and Cheng et al. [50]. All samples were filtered through a 0.22 μm microporous filter membrane before injection to remove impurities from the solution. The chromatographic separation column was Symmetry C 18 (4.6 nm × 250 nm, Column, 5 μm), with a detection wavelength of 280 nm, an injection volume of 10 μL, a column temperature of 25 °C and a flow rate of 1 mL·min^−1^. Gallic acid (GA), ferulic acid (FA), caffeic acid (CA), and p-hydroxybenzoic acid (PHBA) standard samples were purchased from Shanghai Yien Chemical Technology Co., Ltd. (Shanghai, China). They were all chromatographically pure, with a purity of over 98%. The types of phenolic acids in the soil were determined according to the retention time, and the content of phenolic acids in the soil was calculated by the external standard method.

#### 4.4.2. Determination of Physiological and Biochemical Indexes of Seedlings

The 4th–6th healthy, fully expanded leaves from the shoot meristem across all plants of *Z. bungeanum* seedlings were collected, wiped clean, quickly flash-frozen in liquid nitrogen, and brought back to the laboratory to be stored in an ultra-low-temperature refrigerator at −80 °C for testing. The determination of relevant physiological and biochemical indicators was carried out according to the methods of Gao [51] and Li [52]. Among them, malondialdehyde (MDA) content was determined by the thiobarbituric acid method, SOD activity was determined by nitrogen blue tetrazolium photo-reduction method, POD activity was determined by guaiacol reduction method, soluble sugar (SS) was determined by anthrone colorimetric method, the soluble protein (SP) was determined by Coomassie Brilliant Blue (G-250) method, and catalase (CAT) and nitrate reductase (NR) activities were determined by spectrophotometry.

### 4.5. Data Analysis

SPSS 22.0 was applied for statistical analysis and significance test, and multiple comparisons and significance analysis were performed using Duncan’s new multiple range test with a significance level of α = 0.05. Origin 2018 was plotted, and Pearson correlation coefficients between the parameters were analyzed using SPSS. Principal component analysis was also performed using SPSS. All data were expressed as mean ± standard error (Mean ± SE).

## 5. Conclusions

When the volume ratio of microbial fertilizer to bio-organic fertilizer was 2:1, the combined application could significantly reduce the content of phenolic acids (gallic acid and caffeic acid) in the continuous cropping soil of *Z. bungeanum*, improve the antioxidant enzyme activity of the leaves of *Z. bungeanum* seedlings, increase the accumulation of osmoregulatory substances, and reduce the degree of membrane peroxidation, to effectively alleviate the continuous cropping obstacles of *Z. bungeanum*. The results of this study provide some support for sustainable agricultural practices. In the context of the challenges of continuous cropping in agroforestry, our research provides farmers with an environmentally friendly and effective method for soil improvement and crop yield increase. We suggest that farmers use a 2:1 ratio of microbial fertilizer to bio-organic fertilizer in the cultivation of crops susceptible to continuous cropping obstacles, such as *Z. bungeanum*, to improve the soil environment, enhance crop resistance, and promote sustainable agricultural development. At the same time, policy makers should also consider incorporating different types of biofertilizer co-application technologies into agricultural extension programs and encourage farmers to adopt them, to promote the development of agroforestry in a greener and more efficient direction.

## Figures and Tables

**Table 1:** Principal component loadings.

Extracted Ingredients	Eigenvalue	Proportion of Variance (%)	Cumulative Variance (%)
PC1	7.663	69.660	69.660
PC2	1.439	13.080	82.740

**Table 2:** A comprehensive evaluation of the repairing effect of combined application of microbial fertilizer and bio-organic fertilizer on replanting obstacle of *Z. bungeanum.*

Treatments	PC1	PC2	Comprehensive Score	Ranking
T0	−1.41	−0.22	−1.01	8
T1	−0.13	−1.54	−0.29	7
T2	−0.13	0.38	−0.04	4
T3	−0.29	−0.31	−0.24	6
T4	−0.01	−0.97	−0.14	5
T5	−0.16	0.08	−0.10	3
T6	2.07	1.60	1.65	1
T7	0.06	0.98	0.17	2

T0, no fertilization control; T1, 10 g·L^−1^ microbial fertilizer; T2, 100 g·L^−1^ bio-organic fertilizer; T3–T7 denoted that the volume ratio of microbial fertilizer to bio-organic fertilizer was 1:2, 1:4, 1:1, 2:1, and 4:1, respectively.

**Table 3:** Basic physical and chemical properties of continuous cropping soils of *Z. bungeanum.*

pH	Organic Matter (g·kg^−1^)	Total Nitrogen (g·kg^−1^)	Total Phosphorus (g·kg^−1^)	Total Potassium (g·kg^−1^)	Available Nitrogen (mg·kg^−1^)	Available Phosphorus (mg·kg^−1^)	Available Potassium (mg·kg^−1^)
8.29	16.21	0.79	0.61	7.85	45.00	4.36	75.68

## Data Availability

The data that support the findings of this study are available from the corresponding author upon reasonable request.
